# Role of Nitric Oxide, Nitric Oxide Synthase, Soluble Guanylyl Cyclase, and cGMP-Dependent Protein Kinase I in Mouse Stem Cell Cardiac Development

**DOI:** 10.1155/2016/2868323

**Published:** 2016-10-20

**Authors:** Valentina Spinelli, Alessia Vona, Francesca Corti, Lorenzo Diolaiuti, Matteo Zanardelli, Laura Sartiani, Paola Failli

**Affiliations:** ^1^Department of Neurofarba, Pharmacology and Toxicology Unit, University of Florence, Florence, Italy; ^2^Interuniversity Center for Molecular Medicine and Applied Biophysics, Florence, Italy

## Abstract

*Introduction and Aim*. Nitric oxide (NO) can trigger cardiac differentiation of embryonic stem cells (ESCs), indicating a cardiogenic function of the NO synthetizing enzyme(s) (NOS). However, the involvement of the NO/NOS downstream effectors soluble guanylyl cyclase (sGC) and cGMP activated protein kinase I (PKG-I) is less defined. Therefore, we assess the involvement of the entire NO/NOS/sGC/PKG-I pathway during cardiac differentiation process.* Methods*. Mouse ESCs were differentiated toward cardiac lineages by hanging drop methodology for 21 days. NOS/sGC/PKG-I pathway was studied quantifying genes, proteins, enzymatic activities, and effects of inhibition during differentiation. Percentages of beating embryoid bodies (mEBs) were evaluated as an index of cardiogenesis.* Results and Discussion*. Genes and protein expression of enzymes were increased during differentiation with distinctive kinetics and proteins possessed their enzymatic functions. Exogenous administered NO accelerated whereas the blockade of PKG-I strongly slowed cardiogenesis. sGC inhibition was effective only at early stages and NOS blockade ineffective. Of NOS/sGC/PKG-I pathway, PKG-I seems to play the prominent role in cardiac maturation.* Conclusion*. We concluded that exogenous administered NO and other pharmacological strategies able to increase the activity of PKG-I provide new tools to investigate and promote differentiation of cardiogenic precursors.

## 1. Introduction and Aim of the Study

Nitric oxide (NO), a biological active free radical, is an intracellular and intercellular messenger in mammalian cells [1, 2], where its signaling cascade plays an important modulatory role in many physiological and pathological conditions [1, 3]. Besides its well-recognized function in endothelium-derived smooth muscle relaxation [4], synaptic transmission, and immunological responses [5], NO is also a key regulator of heart function via modulation of excitation-contraction coupling [6, 7] and myocardial growth [8].

NO is produced by three different enzymes (namely, neuronal, inducible, and endothelial nitric oxide synthase, nNOS, iNOS, and eNOS) and acts directly or indirectly through the stimulation of the soluble guanylate cyclase (sGC), a heterodimeric enzyme, and the subsequent formation of cyclic GMP (cGMP). Among other direct effects, cGMP activates protein kinase G type I (PKG-I), a serine/threonine kinase exerting many regulatory functions in the heart [9]. On myocardial tissue low activation level of NO/NOS/sGC/PKG-I signaling increases contractility, while high levels exert a negative inotropic effect [7]. In this setting many functional proteins are substrates for PKG-I, including L-type calcium channels and phospholamban (PLB), which in the phosphorylated form relieves its inhibitory effect on sarcoendoplasmic Ca-ATPasi type 2 (SERCA2), enhancing calcium uptake into the sarcoendoplasmic reticulum (SR) and reducing cytosolic free calcium [10, 11].

Increasing evidences point to the participation of reactive oxygen species (ROS), such as NO, hydroxyl radical (OH^−^), hydrogen peroxide (H_2_O_2_), and superoxide anion (O_2_^−^) as important triggers for cardiac differentiation of embryonic stem cells (ESCs) [12, 13]. In stem and progenitor cells, physiological levels of intracellular ROS are required to maintain genomic stability and to increase the proliferation/migration capabilities [14, 15]. Moreover, Cecchi and coauthors [16] demonstrated that ROS as well as NO could be involved in cardiomyogenesis. Additionally, treatment of mouse ESCs (mESCs) with ascorbic acid upregulates NADPH oxidase isoforms, which in turn phosphorylates eNOS and increases cGMP formation leading to enhancing cardiac differentiation from undifferentiated precursors. Interestingly, many studies have shown that NO/NOS/sGC/PKG-I pathway exerts cardioprotective effects in ischemia-reperfusion injury [17], providing the basis to hypothesize that NO/NOS/cGC/PKG-I pathway might be involved in cardiac differentiation of resident stem cell.

In EZ1 mESCs, eNOS (mNos3), and sGC (mGuCy) genes are expressed at early stage of cardiac differentiation [18]. Moreover, treatments of mESCs and human ESCs with NO-donors and sGC activators alone or in combination increase mRNA expression of cardiac specific transcription factor Nkx2.5 and of myosin light chain 2, suggesting a possible cardiogenic role of NO/sGC/PKG-I pathway in undifferentiated precursors [19]. More recently, the NO-donor S-nitrosocysteine proved to enhance cardiac differentiation and spontaneous contraction of D3 mESC [20], corroborating the hypothesis of a cardiogenic function of NO/sGC pathway and that of the downstream effector PKG-I.

To test this hypothesis we used CGR8 mESCs induced to differentiate toward the cardiac lineage and assessed the involvement of the entire signaling pathway NO/NOS/sGC/PKG-I during the differentiation process. Our data provide evidence to support the hypothesis that exogenous administered NO-donors have the potential to promote cardiac differentiation of cardiogenic precursors.

These data have been partially presented as an abstract at the Congress of the European Society of Cardiology, Council on Basic Cardiovascular Science, held in Florence on 8–10 July 2016 [21].

## 2. Material and Methods

### 2.1. Culture of Mouse Embryonic Stem Cells

Mouse embryonic stem cells (mESCs) of the line CGR8 were used [22]. Cell were propagated at 37°C and 5% CO_2_ in gelatin coated dishes, using Glasgow Minimum Essential Medium BHK-21 (G-MEM BHK-21), supplemented with 10% fetal bovine serum (FBS) (GIBCO Invitrogen, Milan, Italy), 2 mM glutamine, 0.1 mM 2-mercaptoethanol, 1 mM sodium pyruvate, 1% nonessential amino acids, 1% penicillin/streptomycin (Sigma Aldrich, Milan, Italy), and 1000 units/mL of Leukaemia Inhibiting Factor (LIF, 10 *μ*g/mL; Millipore, Milan, Italy). All other products for cell cultures were purchased from GIBCO Invitrogen. Prior to splitting, cultures were treated with trypsin/EDTA (Sigma Aldrich) at room temperature for 30–60 sec in order to eliminate differentiated cells. These culture conditions allowed CGR8 mESCs to maintain an undifferentiated phenotype characterized by round-growing, tightly packed, refrangent colonies.

### 2.2. Differentiation of CGR8 ESCs into Embryoid Bodies in Control Condition

CGR8 mESCs were differentiated using the hanging drop method [23]. Drops of differentiating medium containing 600 cells were seeded on the lid of bacteriological dishes. When the dish was closed, this allowed cells to aggregate and grow in suspension (for 2 days) and eventually form mouse embryoid bodies (mEBs). Differentiation medium, in control condition, consisted of medium G-MEM BHK21 supplemented with 10% FBS, 2 mM glutamine, 0.1 mM 2-mercaptoethanol, 1 mM sodium pyruvate, 1% nonessential amino acids, and 1% penicillin/streptomycin. Thereafter, mEBs were resuspended in differentiation medium and grown in suspension in bacteriological dishes for 4 days. On the 6th day, mEBs were plated on gelatin coated dishes, where they attached. After 24–36 h from plating, cardiac differentiation was recognizable from the occurrence of spontaneous contracting areas. mEBs were monitored daily by inverted microscopy; the beating mEBs were counted from day 7 to day 21 (D7–D21). mEBs maturation was followed for no longer than 21 days according to experimental protocols.

### 2.3. Drugs Administration of NO-Donors or NO Pathway Inhibitors during Differentiation of CGR8 ESCs into Embryoid Bodies

In parallel experiments, CGR8 mESCs were differentiated in control conditions and in the presence of the NO-donor or inhibitors of the NO-sGC-PKG-I pathway. Drugs were administered from day 0 (hanging drop formation) to the end of experiments, renewing media every 2 days:

 1,4:3,6-Dianhydro-D-glucitol 5-nitrate (isosorbide mononitrate, ISN, 50 *μ*M, Sigma Aldrich) is a member of the antianginal drug class clinically used to treat Angina Pectoris Prophylaxis, esophageal spasm, and heart failure as a NO-donor.
 1H-[1,2,4]Oxadiazolo[4,3-a]quinoxalin-1-one (ODQ, 1 *μ*M, Sigma Aldrich) is a highly selective, irreversible, heme-site inhibitor of soluble guanylyl cyclase (sGC).
 N^G^-Methyl-L-arginine (L-NMA, 100 *μ*M - Sigma Aldrich) is one of the most commonly used inhibitors of the nitric oxide synthase (NOS).
 KT 5823 (1 *μ*M, Sigma Aldrich) is selective inhibitor of PKG-I.

### 2.4. Molecular Expression of Stemness and Cardiac Specific and NOS-sGC-PKG-I Pathway Genes

*Isolation of Total RNA*,* Reverse Transcription (RT), and Real-Time PCR (Q-PCR)*. Samples of mEBs were collected at different time-points of differentiation for molecular assessment by RNA extraction, reverse transcription, and gene expression analysis using quantitative real-time PCR (Q-PCR).

Total RNA was isolated from mEBs using NucleoSpin® RNA kit (Macherey-Nagel, Düren, Germany). Subsequently, complementary DNA (cDNA) was synthesized from 1 *μ*g total RNA using iScript™ cDNA Synthesis kit (Bio-Rad, Milan, Italy). All of these steps were performed according to the manufacturer's instructions.

Real-time quantitative PCR (Q-PCR) was performed using the default thermocycler program for all genes: 1 minute of preincubation at 95°C followed by 40 cycles for 15 seconds at 95°C, 1 minute at 60°C and 15 seconds at 95°C, 1 minute at 60°C, and 1 minute 95°C. Individual real-time PCR reactions were carried out in 10 *μ*L volumes in a 96-well plate (Applied Biosystems™, London, UK) containing 2 *μ*L RNAse-free water, 1 *μ*L of sense and antisense primers (Bio-Rad), and 5 *μ*L iTaq™ SYBR® Green Universal Supermix (Bio-Rad) plus 2 *μ*L of cDNA sample (5 ng/*μ*L). Each experiment was repeated in triplicate, and quantitative PCR analysis was performed in triplicate and analyzed with Delta Ct-method. Mouse Glyceraldehyde-3-Phosphate Dehydrogenase (mGapdh) was used for internal normalization. Results were analyzed by assuming as 100% the maximal value of gene expression in each experiment. The amplicon context sequences of the primers were listed in Table  1S (see Supplementary Material available online at http://dx.doi.org/10.1155/2016/2868323).

### 2.5. Expression of NOS3, sGC, and PKG-I Proteins during mEBs Maturation

Protein levels were analyzed by western blotting analysis. At different maturation days, mEBs were collected with 100 *μ*L of cold Phosphate-Buffered Saline (PBS) and homogenized in lysis buffer containing 50 mM Tris-HCl pH 8.0, 150 mM NaCl, 1 mM EDTA, 0.5% Triton X-100, and complete protease inhibitor (Roche Diagnostics, Milan, Italy). The homogenate was incubated on ice for 20 minutes. Then, the suspension was sonicated on ice with a wave sonicator for 45 minutes. After centrifugation (12000 ×g for 10 minutes at 4°C), aliquots containing 100 *μ*g (8% of acrylamide for NOS3) or 50 *μ*g total proteins (10% acrylamide for sGC and PKG-I) were blotted on SDS-page. Proteins were revealed using rabbit policlonal anti-NOS3 (eNOS; 1 : 1000; Santa Cruz Biotechnology, Dallas, TX, USA), mouse monoclonal anti-sGC-*β*1 (1 : 1000, Santa Cruz Biotechnology), and rabbit polyclonal anti-PKG-I (1 : 1000, Cells Signalling, Danvers, MA, USA) primary antisera and the corresponding secondary antibodies. Densitometric analysis was performed with the “ImageJ” software and results were normalized to GAPDH immunoreactivity (1 : 1000 rabbit antiserum, Cells Signalling) as internal control. Total protein quantification was performed using bicinchoninic acid assay (BCA method). Bicinchoninic acid and CuSO_4_ solution were purchased from Sigma Aldrich.

### 2.6. NOS and sCG Activity

Cyclic GMP cell content was evaluated as marker of NOS and sGC activity. At day 14 mEBs were washed with warm Dulbecco's Modified Eagle's Medium (DMEM) and incubated with 100 *μ*M 3-isobutyl-1-methylxanthine (IBMX), a nonspecific inhibitor of cAMP and cGMP phosphodiesterases (IBMX-DMEM) for 15 min at 37°C. Then, mEBs were treated according to Table 1 in a total volume of 10 mL.

All drugs were dissolved in IBMX-DMEM and incubation was performed at 37°C. At the end of treatments, mEBs were rapidly frozen and maintained at −20°C until use. cGMP (pg/mg of total proteins) was measured by ELISA kit (Enzo Life Science, Exeter UK). Briefly, mEBs were recovered in 200 *μ*L ice-water and sonicated on ice with a wave sonicator for 30 min and then centrifuged (12000 ×g for 10 minutes at 4°C). Twenty *μ*L of samples was used for protein determination (BCA method). Each sample was acidified (200 *μ*L of HCl 0.1 M, after 15 min at room temperature), hard vortexed, and then centrifuged (12000 ×g for 10 minutes at 4°C). cGMP quantity was measured using ELISA kit according to manufacturer's protocol and absorbance was read in a Wallac Victor spectrophotometer (Perkin Elmer, Milan, Italy). IBMX and S-nitroso-N-acetyl-DL-penicillamine (SNAP, S-nitrosothiol which serves as a NO-donor) were purchased from Sigma Aldrich.

### 2.7. PKG-I Activity

To evaluate PKG-I activity, serine/threonine phosphorylated proteins (containing −1 or +1 phenylalanine/tryptophan residues) were measured. Briefly, at day 14 mEBs were washed with DMEM and incubated with IBMX-DMEM for 15 min at 37°C. Then, mEBs were treated according to Table 2 in a total volume of 10 mL.

All drugs were dissolved in IBMX-DMEM and incubation was performed at 37°C and then mEBs were then rapidly frozen in liquid nitrogen and maintained at −20°C until use. Aliquots of cell proteins (20 *μ*g) were blotted on 10% acrylamide gels and serine/threonine phosphorylated proteins revealed using rabbit anti-phospho-serine/threonine (1 : 1000 rabbit antiserum, Abcam, Cambridge, UK). Densitometric analysis was performed using the “ImageJ” software and results were normalized to GAPDH immunoreactivity as internal control. Unstimulated sample density (GAPDH normalized) was set as 100% in each experiment. Aliquots of same cell proteins (50 *μ*g) were blotted on 10% acrylamide gels and PKG-I was analyzed as already described. The membrane-permeant, stable activator of PKG-I Sp-8-pCPT-PET-cGMPS (cGMPS) was obtained from BIOLOG, Life Science Institute (Bremen, Germany).

### 2.8. Dosage LDH in Conditioned Medium of mESCs

Cells release LDH after tissue damage. Since LDH is a fairly stable enzyme, it has been widely used to evaluate cell damage and toxicity. The mEBs were maintained from day 0 in the presence of 100 *μ*M L-NMA, 1 *μ*M ODQ, 1 *μ*M KT5823, or control conditions and supernatants were collected and frozen at various days of differentiation until use. The amount of the enzyme LDH in mEBs supernatant was evaluated by Lactate Dehydrogenase Activity Assay Kit (Sigma Aldrich) following the manufacturer's directions. NADH was specifically detected by colorimetric assay (450 nm) using Wallac Victor spectrophotometer.

### 2.9. Data Analysis and Statistics

All data are expressed as the mean ± standard error of the mean (s.e.m.). Offline data analysis was performed by using Origin 9.1 (MicroCal Software Inc., MA, USA). Statistical analysis was performed by using ANOVA, followed by Bonferroni's* post hoc* test. A probability value of less than 0.05 was considered as significant.

## 3. Results

### 3.1. mNos3, mGuCy1b, and mPrkg1 in mESCs and during Cardiac Differentiation

**Figure 1:**
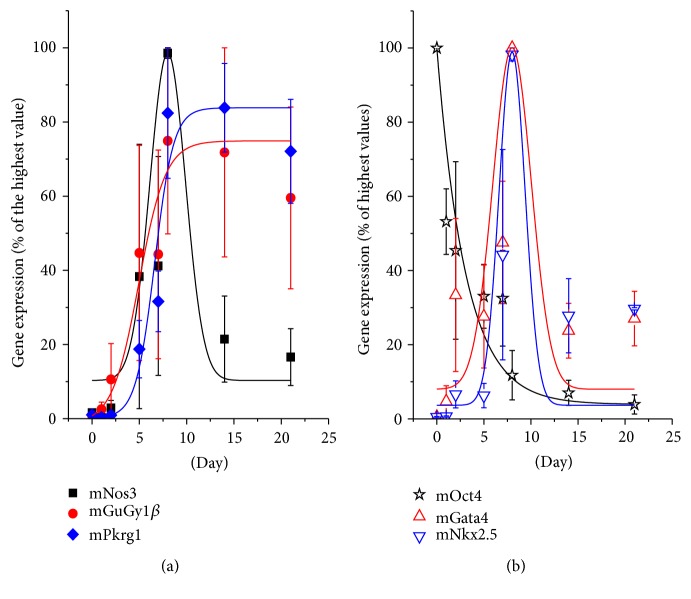
Time-course of gene expression. (a) Relative expression of mNos3, mGuCy1*β*, and mPrkg1 genes. (b) Relative expression of mOct4 (pluripotency marker), mGata4, and mNkx2.5 (cardiac specific) genes. Data are the mean ± s.e. of 3 different differentiations; measures were performed in triplicate.

Gene expression analysis of mNos3, mGuCy1b, and mPrkg1 was performed in undifferentiated mESCs and during cardiac differentiation. Data show that mNos3, mGuCy1b, and mPrkg1 were expressed at very low levels in undifferentiated cells, but they collectively increased during cardiac differentiation, albeit with different time-courses. mNos3 (Figure 1(a)) showed a bell-shaped curve, with a robust raise at day 5 and a peak at day 8, a key developmental period in* in vitro* cardiogenic process. mGuCy1b and mPkg1 increased robustly at day 5, reached the maximum at day 8, and maintained these expressions until the end of the maturation process (day 21).

In the same preparations, we monitored cardiac differentiation of mESCs, analyzing gene expression profile of pluripotency (mOct4 and mNanog), early mesodermic (mBrachyury) and mature cardiac marker (mNkx2.5, mMef2c and mGata4) genes. Following the process, the pluripotency transcription factor mOct4 was clearly downregulated, as expected, showing a specular time-course compared to mPrkg1, mGuCy1b, and mNos3 in the first phase (Figure 1(b)). Similarly to mOct4, mNanog expression steadily dropped (Figure  1S) during the differentiation; conversely, cardiac transcription factors mNkx2.5 (Figure 1(b)), mGata4 (Figure 1(b)), and mMef2c (Figure 1S) were progressively upregulated from day 0 to day 8 and then declined, following a bell-shaped curve that is almost superimposable to that of mNos3. The mesodermal marker mBrachyury showed a transient expression pattern peaking at day 2 (Figure 1S).

### 3.2. NOS3, sCG, and PKG-I Protein Expression

**Figure 2:**
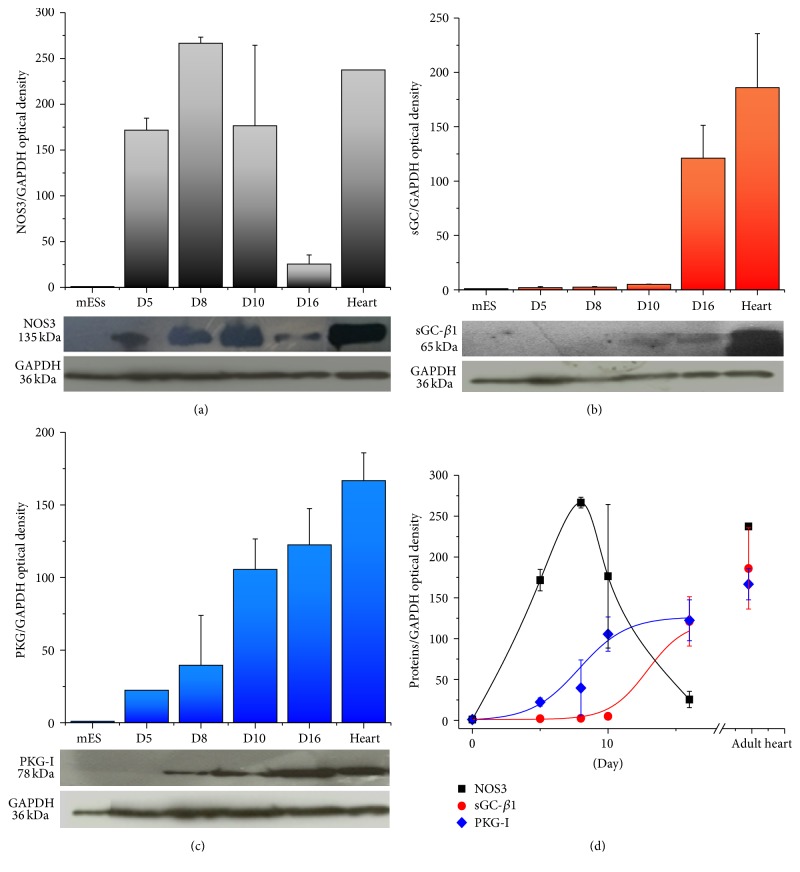
NOS3, sGC-*β*1, and PKG-I protein expression in mESCs, in differentiating mEBs and in adult mouse hearts. Protein expression of NOS3 (a), sGC-*β*1 (b), and PKG-I (c) in mESCs and mEBs at days 5, 8, 10, and 16 of differentiation and in the adult mouse heart. Representative images of gels are shown. (d) It shows time-courses of all proteins. Data are the mean ± s.e. of at least 3 differentiations.

At different stages of cardiac differentiation, NOS3, sCG, and PKG-I protein levels were evaluated by western blot analysis. Results are shown in Figures 2(a), 2(b), and 2(c). All enzymes were undetectable in mESCs; NOS3 and PKG-I proteins appeared at day 5 of differentiation, while sGC expression was detected later. NOS3 maximal level was observed at day 8 and then declined, being well detectable at day 10 and essentially absent at day 16 (Figure 2(a)). sGC-1*β* was evident at day 10 and rapidly increased until day 16 (Figure 2(b)), showing similar values at day 12. Finally, PKG-I showed a slower, progressive increase during the differentiation process that reached a plateau level at day 10 and remained constant until day 16 (Figure 2(c)).  Figure 2(d) shows the superimposition of protein time-courses during the entire differentiation process.

### 3.3. Soluble GC and NOS Enzymatic Activities

**Figure 3:**
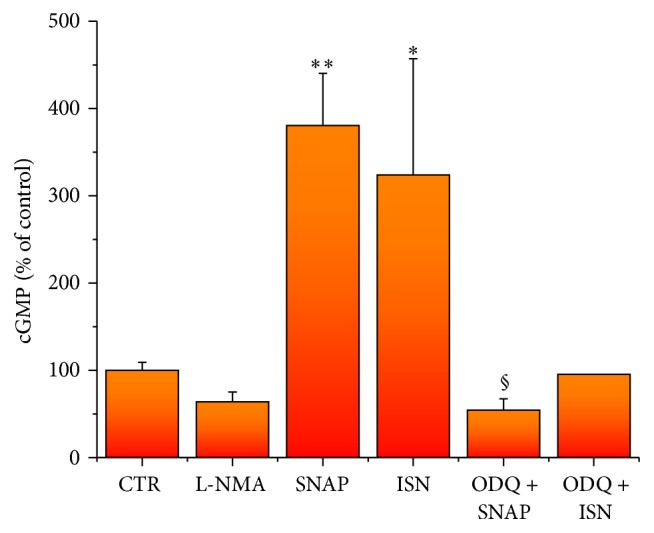
Soluble GC and NOS enzymatic activity. Soluble GC and NOS enzymatic activity evaluated by cGMP measure in mEBs at day 14 of differentiation in control condition and in the presence of 100 *μ*M L-NMA, 100 *μ*M SNAP, 50 *μ*M ISN, 1 *μ*M ODQ + 100 *μ*M SNAP, and 1 *μ*M ODQ + 50 *μ*M ISN. All measures were carried out in the presence of 100 *μ*M IBMX. ^*∗*^ *P* < 0.05 and ^*∗∗*^ *P* < 0.01 versus CTR; ^§^ *P* < 0.05 versus SNAP, one-way ANOVA followed by* post hoc* Bonferroni's test. For method, see Table 1. Data are the mean ± s.e. of at least 3 differentiations; measures were performed in duplicate.

To assess sGC and NOS enzymatic activities, we estimated cGMP production in different experimental conditions at day 14, a time-point when both proteins are clearly expressed (Figure 3).

After 60 min incubation with IBMX, in control condition cGMP concentration was 0.84 ± 0.187 pmol/mg of total proteins. After incubation with L-NMA, an inhibitor of NOS, cGMP quantity was slightly decreased to about 60%, suggesting that in control condition NOS basal activity is able to induce a modest, albeit detectable, sGC stimulation. SNAP or ISN, two NO-donors, significantly increased cGMP levels more than 3 times (3.80 and 3.25, resp.) compared to control. When either SNAP or ISN was added in the presence of the sGC inhibitor ODQ, increase of cGMP was significantly (SNAP) or strongly (ISN) prevented, giving values not different from control.

### 3.4. PKG-I Enzymatic Activity

**Figure 4:**
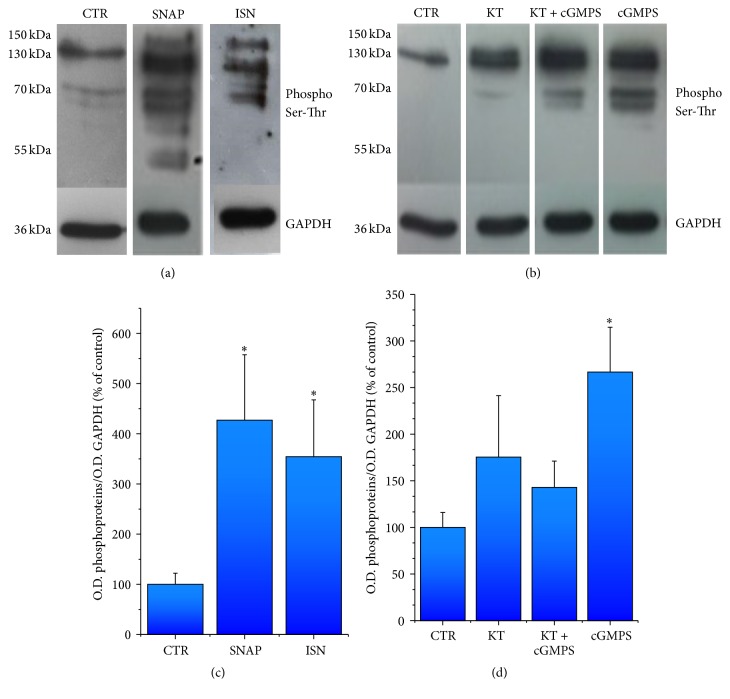
PKG-I activity; it was evaluated by serine/threonine phosphorylated proteins in mEBS at day 14 of differentiation in control condition and in presence of 100 *μ*M SNAP, 50 *μ*M ISN, 1 *μ*M KT5823, 1 *μ*M KT5823 + 1 *μ*M cGMPS, and 1 *μ*M cGMPS. All measures were carried out in the presence of 100 *μ*M IBMX. Representative images of gels are shown in (a) and (b). ^*∗*^ *P* < 0.05 versus control, one-way ANOVA followed by* post hoc* Bonferroni's test. For method, see Table 2. Data are the mean ± s.e. of at least 3 different differentiations.

PKG-I activity was evaluated by western blot analysis of phosphorylated proteins. Figures 4(a) and 4(b) show representative panels of phosphorylated proteins obtained in different experimental conditions, which were analyzed by semiquantitative analysis (Figure 4(c)). Both NO-donors (SNAP and ISN) significantly increased protein phosphorylation compared to control; similarly, the cGMP membrane permeable cGMPS significantly increased protein phosphorylation by 250% (Figure 4(d)). The PKG-I inhibitor KT 5823 did not modify significantly protein phosphorylation compared to basal conditions, while it significantly prevented by 50% the phosphorylation induced by cGMPS (Figure 4(d)).

PGK-I protein expression levels detected in each experimental sample used to quantify phosphorylation were very similar (Figure 2S), ruling out the possibility that differences in phosphorylation could be dependent on PKG-I expression variability.

### 3.5. Functional Assessment of the Effect of NO-Donor or NOS, sGC, and PKG-I Inhibition on Spontaneous Beating of Differentiating Embryoid Bodies

**Figure 5:**
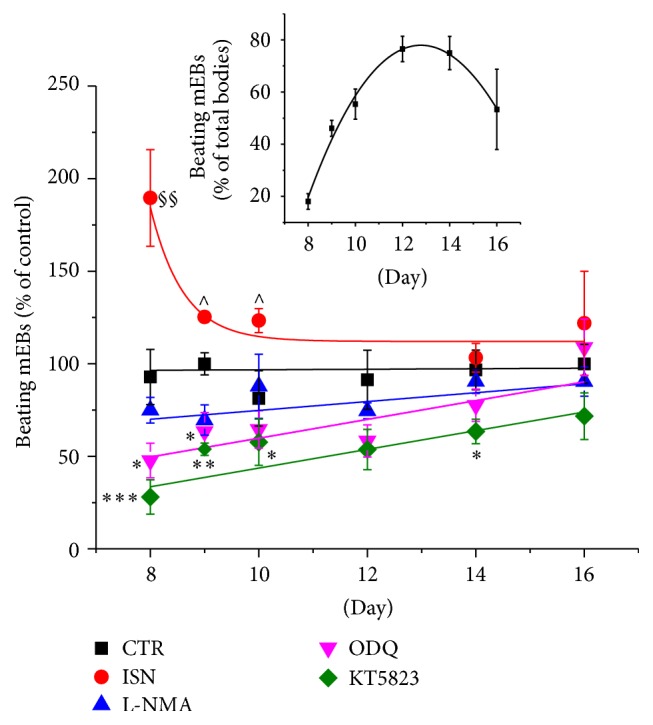
Percentage of beating mEBs during cardiac differentiation. The percentage of beating mEBs was calculated at different time-points of mEBs cardiac differentiation. Cells were incubated with 50 *μ*M ISN (NO-donor) or NOS/sGC/PKG-I pathway inhibitors (resp., 100 *μ*M L-NMA, 1 *μ*M ODQ, or 1 *μ*M KT5823) from day 0 to day 16. Insert: beating mEBs (percentage of the total bodies) in control condition. One-way ANOVA followed by* post hoc* Bonferroni's test. ^*∗∗∗*^ *P* < 0.001, ^*∗∗*^ *P* < 0.01, and ^*∗*^ *P* < 0.05 versus control; ^§§^ *P* < 0.01 versus control and all other treatments; ^∧^ *P* < 0.05 versus all other treatments. Data are the mean ± s.e. of at least 4 different differentiations.

To assess the impact of NO/NOS/sGC/PKG-I pathway on a functional marker of cardiac differentiation, we evaluated the percentage of beating embryoid bodies in different experimental conditions. As expected, in control condition beating increased from day 8 of differentiation, reaching a maximal value at day 12 (Figure 5, insert).

In parallel experiments, we assessed the effect exerted by the NO-donor ISN, the NOS-inhibitor L-NMA, the sGC inhibitor ODQ, or the PKG I-inhibitor KT5823, setting control values of each day as 100% (Figure 5). In the presence of ISN, percentage of beating increased significantly compared to control evaluated at day 8 (*P* < 0.001 versus control and other treatments), while it was slightly higher at day 9 (*P* < 0.05 versus other treatments) and at day 10 (*P* < 0.05 versus control and other treatments). Thereafter, values became undistinguishable from control. ODQ reduced mEBs beating at days 8 and 9 (*P* < 0.05); then the difference disappeared. KT5823 strongly reduced beating at day 8 (*P* < 0.001) and maintained this inhibitory effect constant. Inhibition of NOS did not significantly reduce beating at any stage of maturation (Figure 5).

### 3.6. Lactate Hydrogenase Activity

Since the reduction of the contractile activity observed in the presence of NO-sGC-PKG-I inhibitors could mirror a latent toxicity of the selected drugs, we performed the dosage of LDH as a marker of a specific cell toxicity. LDH activity was 3.01 ± 0.604 mU/mL in control conditions at day 4. This value remained similar, independently of the maturation day. LDH activity was increased in the supernatant of mEBs treated with ODQ at day 4, but then it returned to control value, indicating a relative toxicity caused by ODQ at the beginning of the treatment, which however disappeared shortly thereafter. All the other treatments did not significantly influence LDH activity. Results are depicted in Figure 3S.

## 4. Discussion

Assessment of cardiac differentiation* in vitro* is a prerequisite to identify factors and drugs able to increase the cardiogenic potential of stem cells not only during organogenesis, but also during the healing and reparation after degenerative processes of the heart. In particular, after cardiac ischemic insults, the self-reparative program is limited, thus requiring additional studies to identify new effective therapies and/or novel mechanistic insights into the action of drugs able to potentiate the healing process. Among others, NO-donors are a treatment of choice for ischemic and postischemic insult. In the heart, endogenous and exogenous NO acts through activation of sGC that produces cGMP, which in turn increases the phosphorylation activity of PKG-I, a final effector enzyme responsible for complex regulatory function of cardiac contractility [7]. Whether the function of NO/NOS/cGMP/PKG-I pathway also extends to the cardiogenic process of stem cells is only partially known. Therefore, in our study we investigated thoroughly the involvement of the pathway in the cardiac differentiation of mESCs, directing our efforts to the physiological and pharmacological role of NO (endogenously produced by NOS3 or exogenously administered as ISN) and of the main downstream effectors sGC and PKG-I.

During the cardiogenic process of CGR8 ESC, our data showed that expression of mNos3, mGuCy1b, and mPkrg1 parallels the increase of cardiac specific gene pattern and the decrease of pluripotent genes (Oct4 and Nonog). In particular, mNos3 gene maximal expression was observed at day 8, when cardiac specific genes reach their maximal expression (Figure 1(b)). Thereafter, gene expression levels declined and remained stable throughout the process. Interestingly, our data on eNOS time-course of expression in differentiating CGR8 mESCs are in line with that detected in mESCs on ascorbic acid treatment [24]. At variance with eNOS, mGuCy and mPkrg1 expression appeared delayed and remained stably high throughout the developmental period. These data confirm the results already reported for the cardiac differentiation of a different cell line, the EZ1 mouse ESC [18], where a similar temporal pattern of Nos3/sGuCy expression was detected. Of note, our investigation extends the transcript analysis to mPkrg1, the downstream enzyme of the pathway, responsible for amount of phosphorylation of target proteins. More importantly, our study provides evidence that the entire protein panel comprised in the NO pathway (NOS3-sGC-PKG-I) is functionally expressed in the cardiogenic system; it exhibits a developmental time-course that closely resembles that of the relative primary transcripts, thus suggesting a complete translation machinery in the differentiating system. Indeed, NOS3 protein was readily detectable at the beginning of the differentiation, while its expression decreases in the late developmental phase. sGC was maximally expressed at day 16 of differentiation, later than the respective gene, even if small quantities of the protein were already detectable at day 10. PKG-I was clearly detectable starting from day 5; it increased soon after day 8 and maintained this level until the end of the differentiation process. Functionally, all enzymes possess their typical activities, as determined by measuring directly or indirectly their metabolic or functional products at day 14. Accordingly, cGMP quantities were enhanced by stimulating sGC with NO-donors and reduced by blocking NOS activity. Moreover, PKG-I mediated phosphorylation capacity was increased by NO-donors and cGMP stable analog, showing a full functional maturation of the enzymatic system attained during cardiac differentiation. A similar time-course of NOS3-sGC-PKG-I protein expression has been reported for EZ1 mouse ESCs [18], where sGC activity and its enhancement by NO-donors were measured at day 14. At variance with our study, sGC in EZ1 mouse ESCs was detectable from day 5 to day 7, a discrepancy likely dependent on the specific cell line.

One of the indices of cardiac lineage specification is the development of spontaneous beating areas in mEB, a requisite that in CGR8 ESCs typically appear 7-8 days after differentiation onset and then peak around day 12. We observed that administration of exogenous NO or endogenous cGMP/PKG-I pathway blockade is able to change the number and timing of beating areas in opposite directions. Of note, the increased beating observed after exogenous NO treatment at the beginning of the differentiation (day 8, Figure 5) suggests that the stimulation of NO pathway may promote cardiac differentiation of ESCs. This hypothesis is corroborated by similar findings observed by Hodge and coauthors [20] in a different line of genetically modified mESCs, where exposure to S-nitrosocysteine, an exogenous NO-donor, increased the number of contracting areas detected at early phase of differentiation (10th day). In the same setting, inhibition of endogenous NO production via NOS blockade was ineffective in reducing the percentage of contracting areas [20]. Accordingly, a different study reported that NOS inhibition in basal condition was unable to influence the spontaneous beating percentage of mEBs [24]. Our data extend the above results providing evidence that NO downstream effectors sGC and PKG-I are also importantly involved in the cardiogenic process. Indeed, in the presence of ODQ or KT 5823, sGC, and PKG-I inhibitors, respectively, appearance of the contractile phenotype was substantially delayed (Figure 5), leading to concluding that the entire signaling pathway is necessary to complete the cardiogenic process. Interestingly, sGC inhibition was able to reduce the percentage of beating mEBs only at the beginning of the differentiation (i.e., until day 10), when, though sGC protein expression is low, its functional activity is sufficient to allow beating appearance, as revealed by ODQ effect. Since sGC inhibition was effective to delay beating appearance, whereas blockade of endogenous NO production did not produce any evident effect, we hypothesize that other endogenous activators of sGC, such as carbon monoxide (CO), might be involved in the differentiation process. CO is an endogenous activator of sGC with a 50 time lower efficacy than NO [25], produced by heme oxygenase 1 and heme oxygenase 2. Our preliminary data show that heme oxygenase 1 and 2 genes are expressed in parallel with NO pathway enzymes during cardiac differentiation of CGR8 ESCs, thus corroborating the idea that CO may represent a potential candidate for sGC activation in this setting. Considering that heme oxygenase 1 upregulation plays a protective role in cardiac stem cell apoptosis [26], we can hypothesize that in our experimental condition CO could reinforce or substitute NO in sGC stimulation, a key enzyme in cardiac maturation. Further research is necessary to clarify the role of CO and heme oxygenase in ESC biology and cardiac differentiation.

Our data demonstrate that PKG-I blockade strongly delays the development of spontaneous beating areas throughout the whole differentiation process at variance with the limited effect of sGMP inhibition, a finding that suggests a predominant role of PKG-I over sGMP during the cardiogenic process. This leading function of PKG-I on cardiogenic differentiation agrees with the notion that a basal activity is also present at very low cGMP levels, which are only sufficient to provide a minimal enzymatic activity [27]. Furthermore, a central role of the PKG-I activity in the cardiogenic process is also demonstrated by tadalafil treatment, a cGMP-specific phosphodiesterase 5A (PDE5A) inhibitor, which proved to promote survival and proliferation of mesenchymal stem cells in a rat model of infarction [28]. Even if obtained in a different model, these data indicate an important role of the PKG-I activity shared by different stem precursors, to enhance the cardiogenic potential.

Our findings demonstrate that blockade of NOS-cGMP-PKG-I pathway in developing mEBs is not associated with release of LDH in the supernatant; this suggests that the blockade of a specific program and not an unspecific toxicity is likely the mechanism that suppresses cardiac differentiation.

In our study we did not assess the molecular mechanism linking PKG-I activity to cardiogenic process. This issue needs further studies besides the aims of present investigations; however some interesting clues may be drawn considering the role of PKG-I in intracellular Ca^2+^ homeostasis. At early differentiation stages, spontaneous contractions of differentiating ESCs are dependent on spontaneous cytosolic free Ca^2+^ oscillations. These latter mainly depend on SR discharge and subsequent reuptake. Indeed, mRNAs/proteins that characterize the mature functional SR in adult cardiac myocytes (type 2 ryanodine receptor, junctophilin-2, calsequestrin, PLB, and SERCA2a) are already expressed at early stages of differentiating ESCs and well before the appearance of spontaneous contractions [29]. Additionally, in the same setting functional inositol-1,4,5-trisphosphate receptor (IP3R) channels proved to contribute to Ca^2+^ rhythmic discharge from SR [30], meaning that a complex network of Ca^2+^ regulating proteins is already functionally expressed in differentiating ESCs. Of note, in adult cardiac cells all of these proteins are known targets of PKG-I activity [9], which thus exert a fundamental regulatory function of intracellular Ca^2+^ homeostasis. In particular, PKG-I phosphorylates PLB and enhances Ca^2+^ reuptake into SR by accelerating SERCA2a activity [10, 11]; this mechanism increases SR Ca^2+^ content that might accelerate the in/out Ca^2+^ cycling between SR and cytosol. PKG-I also targets sodium/calcium exchanger (NCX), which in turn contributes to its reverse mode to cytosolic Ca^2+^ oscillations. Finally, sGC/PKG-I pathway is reported to activate cytosolic Ca^2+^ influx via reverse mode NCX activity [31]. In our setting, we hypothesize that the growing functional expression of PKG-I during differentiation, through modulation of cytosolic Ca^2+^ cycling, may favor the induction of Ca^2+^ discharge from SR and the progression of the cardiogenic process.

In conclusion, our research supports the idea that PKG-I pathway is a key promoter to drive ES cell differentiation in cardiac cell. Though further research is necessary, these findings suggest that treatments able to increase PKG-I activity* in vivo* (i.e., NO/CO-donors, direct activator of sGC, and PDE5 inhibitors) could be effective in stimulating resident cardiac stem cells to differentiate in mature cardiac cells, favoring cardiac healing and ameliorating the ischemic insult. A thorough preclinical assessment of survival, proliferation, and cardiac differentiation of stem cells will clarify the plausibility of this hypothesis and will shed novel light into the benefits of NO-donors in cardiac ischemia and other degenerative pathologies.

## Supplementary Material

Table 1S: Isolation of total RNA, Reverse Transcription (RT) and Real Time PCR (Q-PCR). Samples of mEBs were collected at different time-points of differentiation for molecular assessment by RNA extraction, reverse transcription, and gene expression analysis using quantitative real-time PCR (Q-PCR) and real-time quantitative PCR (Q-PCR) was performed. The amplicon context sequences of the primers were listed in Table 1S.
Figure 1S: Gene expression profile of pluripotency (mNanog), early mesodermic (mBrachyury) and mature cardiac marker (mMef2c) genes in mESCs and during cardiac differentiation. Gene expression analysis of pluripotency (mNanog), early mesodermic (mBrachyury) and mature cardiac marker (mMef2c) was performed in undifferentiated mESCs and during cardiac differentiation. During the differention, the pluripotency transcription factor mNanog steadily dropped; mMef2c was progressively up-regulated from day 0 to day 8 and then declined, following a bell-shaped curve. The mesodermal marker mBrachyury, showed a transient expression pattern peaking at day 2.
Figure 2S: PGK-I protein expression levels of samples used to quantify serine/threonine phosphorylation by PKG-I. PGK-I protein expression levels were detected in each experimental sample used to quantify serine/threonine phosphorylation by PKG-I. As shown in figure 2S, western blot analysis confirmed that PKG-I expression level was very similar in each sample, ruling out the possibility that differences in phosphorylation could be dependent on PKG-I expression variability.
Figure 3S: Lactate hydrogenase activity. LDH activity was 3,01± 0,604 mU/ml in control conditions at day 4. This value remained similar, independently of the maturation day. LDH activity was increased in the supernatant of mEBs treated with ODQ at day 4, but then it returned to control value, indicating a relative toxicity caused by ODQ at the beginning of the treatment, which however disappeared shortly thereafter. All other treatments did not significantly influenced LDH activity.

## Figures and Tables

**Table 1:** mEBs incubation time and drug administration.

Groups	15 min	30 min
CTR	IBMX-DMEM	IBMX-DMEM
100 *μ*M L-NMA	L-NMA	IBMX-DMEM
100 *μ*M SNAP	IBMX-DMEM	SNAP
50 *μ*M ISN	IBMX-DMEM	ISN
1 *μ*M ODQ + 100 *μ*M SNAP	ODQ	SNAP
1 *μ*M ODQ + 100 *μ*M ISN	ODQ	ISN

**Table 2:** mEBs incubation time and drug administration.

Groups	15 min	10 min
CTR	IBMX-DMEM	IBMX-DMEM
1 *μ*M KT5823	KT5823	IBMX-DMEM
1 *μ*M cGMPS	IBMX-DMEM	cGMPS
1 *μ*M KT5823 + 1 *μ*M cGMPS	KT5823	cGMPS
100 *μ*M SNAP	IBMX-DMEM	SNAP
50 *μ*M ISN	IBMX-DMEM	ISN
