# Supplementary material for: Role of Nitric Oxide, Nitric Oxide Synthase, Soluble Guanylyl Cyclase, and cGMP-Dependent Protein Kinase I in Mouse Stem Cell Cardiac Development
Source: Stem Cells Int. 2016 Oct 20;2016:2868323. doi: 10.1155/2016/2868323 (PMC5093303; doi:10.1155/2016/2868323)
Supplement: Supplementary file 1 — Table 1S: Isolation of total RNA, Reverse Transcription (RT) and Real Time PCR (Q-PCR). Samples of mEBs were collected at different time-points of differentiation for molecular assessment by RNA extraction, reverse transcription, and gene expression analysis using quantitative real-time PCR (Q-PCR) and real-time quantitative PCR (Q-PCR) was performed. The amplicon context sequences of the primers were listed in Table 1S. Figure 1S: Gene expression profile of pluripotency (mNanog), early mesodermic (mBrachyury) and mature cardiac marker (mMef2c) genes in mESCs and during cardiac differentiation. Gene expression analysis of pluripotency (mNanog), early mesodermic (mBrachyury) and mature cardiac marker (mMef2c) was performed in undifferentiated mESCs and during cardiac differentiation. During the differention, the pluripotency transcription factor mNanog steadily dropped; mMef2c was progressively up-regulated from day 0 to day 8 and then declined, following a bell-shaped curve. The mesodermal marker mBrachyury, showed a transient expression pattern peaking at day 2. Figure 2S: PGK-I protein expression levels of samples used to quantify serine/threonine phosphorylation by PKG-I. PGK-I protein expression levels were detected in each experimental sample used to quantify serine/threonine phosphorylation by PKG-I. As shown in figure 2S, western blot analysis confirmed that PKG-I expression level was very similar in each sample, ruling out the possibility that differences in phosphorylation could be dependent on PKG-I expression variability. Figure 3S: Lactate hydrogenase activity. LDH activity was 3,01± 0,604 mU/ml in control conditions at day 4. This value remained similar, independently of the maturation day. LDH activity was increased in the supernatant of mEBs treated with ODQ at day 4, but then it returned to control value, indicating a relative toxicity caused by ODQ at the beginning of the treatment, which however disappeared shortly thereafter. All other treatments [file 2868323.f1.pdf]

Table 1S – Amplicon context sequences of primers used for Q-PCR

Mouse Glyceraldehyde-3-Phosphate Dehydrogenase (mGapdh)	TGGGAGTTGCTGTTGAAGTCGCAGGAGACAACCTGGTCCT CAGTGTAGCCCAAGATGCCCTTCAGTGGGCCCTCAGATGC CTGCTTCACCACCTTCTTGATGTCA
Mouse Octamer-binding Transcription factor 4 (mOct4)	ATGCAAATCGGAGACCCTGGTGCAGGCCCGGAAGAGAAA GCGAACTAGCATTGAGAACCGTGTGAGGTGGAGTCTGGAG ACCATGTTTCTGAA
Mouse Brachyury (mBrachyury)	CCACCGGTCATCGCCCTACCCCAGCCCCTATGCTCATCGGA ACAGCTCTCCAACCTATGCGGACAATTCATCTGCTTGTCTG TCCATGCTGCAGTCCCATGATAACTGGTCTAGCCTCGGAGT
Mouse Myocyte Enhancer factor 2C (mMef2c)	TTCAGATTACGAGGATAATGGATGAGCGTAACAGACAGGT GACTTTTACGAAGAGGAAATTTGGATTGATGAAGAAGGCT TATGAGCTGAGCGTGCTGTGCGACTGTG AGATTGCACTG
Mouse GATA binding protein 4 (mGata4)	CTGGGACATGGAGCTGCTGTGCCCATAGTGAGATGACAGC CCGGGCTCTGTCTTGATGGGGCGCATCTCTTCACTGCTGCT GCTGCTGCTAGTGGCATTGCTGGAGTTACCGCTGGAGGCA CCACTGGAGGGAGGGAGGGTCTCACCAGCAGGACCTGCT GGCGTCTTAGATTTATTCAGGTTCTTGGGCTTCCGTTTTCTG GT
Mouse NK2 transcription factor related, locus 5 (Drosophila), (mNkx2.5)	GTGGTCTCTCGGCGCCATCCGTCTCGGCTTTGTCCAGCTCC ACTGCCTTCTGCAGCGCGCACAGCTCTTTTTTATCCGCCCG AGGGTCTTTGGCTGGGTCAGGGTCACCGTAGGCTCCCGGG TAAAATGTGGGGGCGGCTGGGAAAGCAGGAGAGCACTTG GGGGGCGAAGGCGCG
Mouse Nanog homeobox (mNanog)	TCCAGCAGATGCAAGAACTCTCCTCCATTCTGAACCTGAGC TATAAGCAGGTTAAGACCTGGTTTCAAAACCAAAGGATGA AGTGCAAGCGGTGGCAGAAAAACCAGTGGTTGAAGACTA GCAATGGTCTGATTCAGAAGGGCT
Mouse Nitric Oxide Synthase 1 (neuronal) (mNos1)	GGCAACCATGAGGACCTCGTGAATGCACTCATTGAACGGC TGGAGGACGCGCC GCCTGCCAACCACGTGGTGAAGGTGGAAATGCTGGAGGAA AGGAACACGGCTC TGGGTGTCATCAGTAATTGGAAGGATGAATCTCGCCTCC
Mouse Nitric Oxide Synthase 2 (inducible) mNos2	ACCACACCAAACTGTGTGCCTGGAGGTTCTGGATGAGAGC GGCAGCTACTGGG TCAAAGACAAGAGGCTGCCCCCCTGCTCACTCAGCCAAGC CCTCACCTACTTCC TGGACATTACGACCCCTCCCACCC
Mouse Nitric Oxide Synthase 3 (endothelial) (mNos3)	GTCCTGTGCATGGATGAGTATGATGTGGTGTCCCTAGAGC ACGAGGCACTGGTGTTGGTGGTGACCAGCACATTTGGCAA TGGGGATCCTCCGGAGAATGGAGAGAGCTTTGCAGCAGCG CTCATGGAAATGTCAGGCCCGTACAACAGCTCCCCTAGGC CTGAGCAGCACAAGAGCTACAAAATCCGATTCAACAGTGT CTCCTGCTCAGACCC ACT
Mouse Protein Kinase, cGMP-dependent, type I (mPrkg1)	TATCATGTCAATCCCCCGCAGTATGATATTGTAGGTTTTCA TTGGATCTGGGCCT GAGAAAGGTGGGCTGCCAGTCAGAAGCTCATACATCAGAA TTCCTAGTGACCA
Mouse Guanylate cyclase 1, soluble, beta (mGuCy1b)	AGGCCAGCCAGTATGTAGAGAGCATGTTCCGAGCCTTCGA CAAGAATGGGGACA ACACCATTGACTTCCTGGAGTATGTGGCAGCCCTGAACCTC GTGCTG

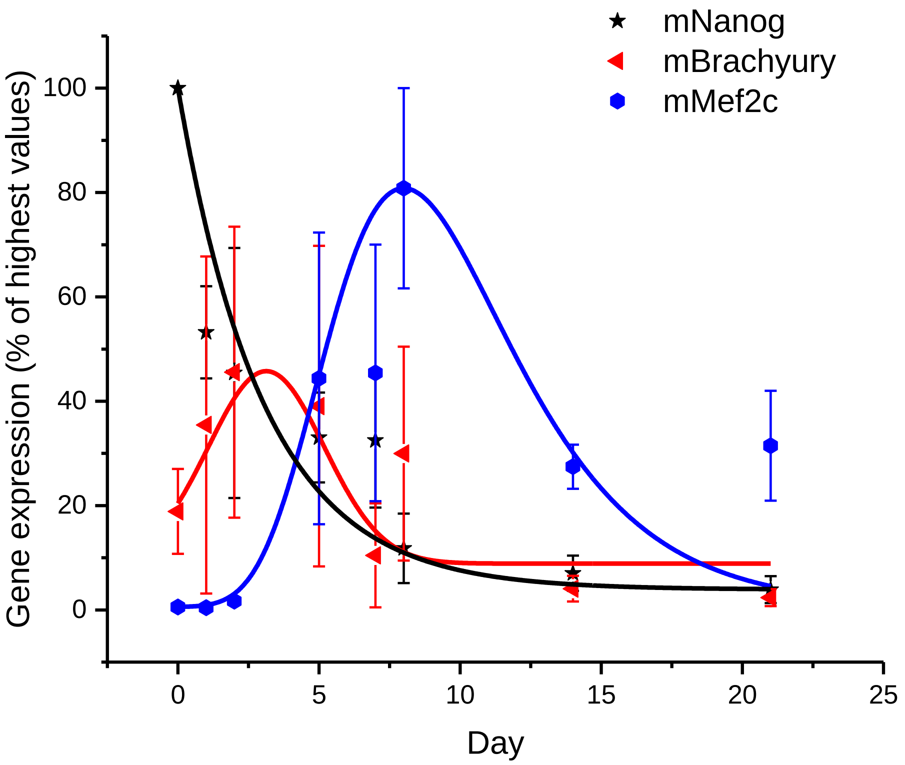

Figure 1S. Time-course of gene expression in differentiating mEBs - Relative expression of mNanog (pluripotency marker), mBrachyury (early mesodermic) and mMef2c (cardiac specific) genes. Data are the mean ± s.e. of 3 differentiations; measures were performed in triplicate.

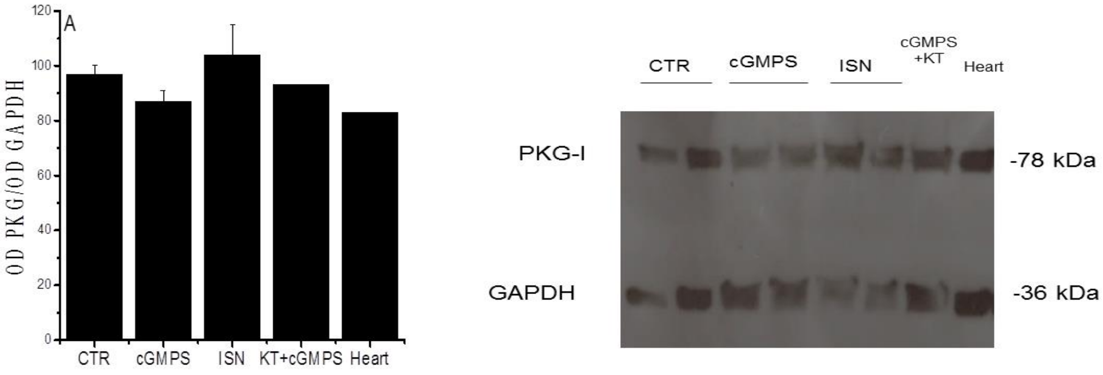

Figure 2S - PGK-I protein expression in a single experimental preparation used to quantify protein phosphorylation (see figure 4). Different drug treatments were performed as described in the method (scheme 2).

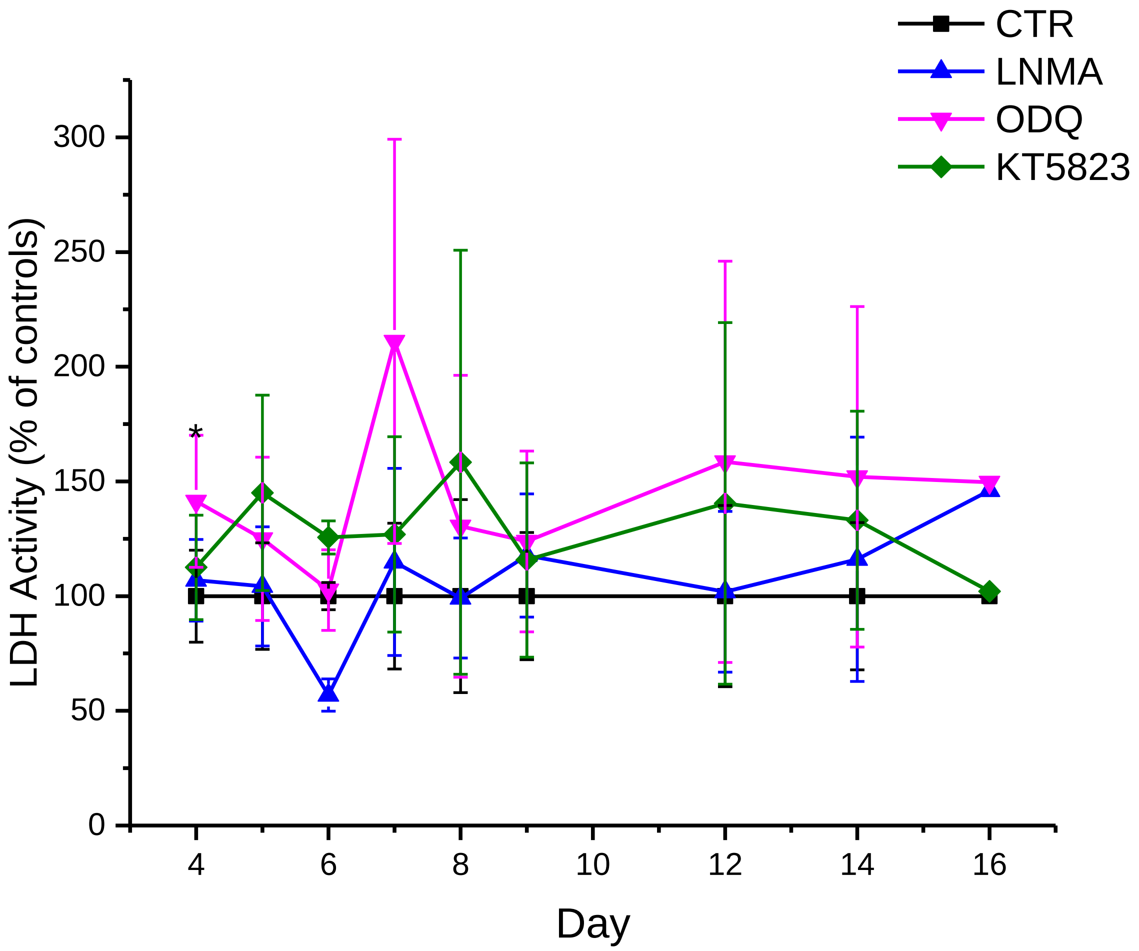

Figure 3S - LDH release during mEBs cardiac differentiation - LDH activity in conditioned medium of mEBs was measured in control conditions or in the presence of either 50 μM ISN (NO-donor) or NOS/sGC/PKG-I pathway inhibitors (respectively, 100 μM L-NMA, 1 μM ODQ or 1 μM KT5823) from day 0 to 16. Data are expressed as percentage of control values. *P<0.05 vs control, one way ANOVA followed by post-hoc Bonferroni's test**.** Data are the mean ± s.e. of at least 3 different differentiations; measures were performed in duplicate.
